# Adjustment of oral diet based on flexible endoscopic evaluation of swallowing (FEES) in acute stroke patients: a cross-sectional hospital-based registry study 

**DOI:** 10.1186/s12883-019-1499-8

**Published:** 2019-11-12

**Authors:** Tobias Braun, Martin Juenemann, Maxime Viard, Marco Meyer, Iris Reuter, Mario Prosiegel, Manfred Kaps, Christian Tanislav

**Affiliations:** 1 Department of Neurology, University Hospital Giessen and Marburg, Klinikstrasse 33, 35392 Giessen, Germany; 2 Department of Neurology, Justus Liebig University, Klinikstrasse 33, 35392 Giessen, Germany; 3 Department of Neurology/Geriatrics, Diakonie Klinikum Jung-Stilling, Wichernstraße 40, 57074 Siegen, Germany; 4 Lecturer at Faculty of Languages and Literatures, Department I, Ludwig-Maximilians-University (LMU), Munich, Germany

## Abstract

**Background:**

Diagnosing dysphagia in acute stroke patients is crucial, as this comorbidity determines morbidity and mortality; we therefore investigated the impact of flexible nasolaryngeal endoscopy (FEES) in acute stroke patients.

**Methods:**

The FEES investigation as performed in acute stroke patients treated at a large university hospital, allocated as a standard procedure for all patients suspected of dysphagia. We correlated our findings with baseline data, disability status, pneumonia, duration of hospitalisation, necessity for mechanical ventilation and treatment on the intensive care unit. The study was designed as a cross-sectional hospital-based registry.

**Results:**

We investigated 152 patients. The median age was 73; 94 were male. Ischemic stroke was diagnosed in 125 patients (82.2%); 27 (17.8%) suffered intracerebral haemorrhage.

Oropharyngeal dysphagia was diagnosed in 72.4% of the patients, and was associated with higher stroke severity on admission (median NIHSS 11 [IQR 6–17] vs. 7 [4–12], *p* = .013; median mRS 5 [IQR 4–5] vs. 4 [IQR 3–5], *p* = .012). Short-term mortality was higher among patients diagnosed with dysphagia (7.2% vs. 0%, *p* = .107). FEES examinations revealed that only 30.9% of the patients had an oral diet appropriate for their swallowing abilities.

A change of oral diet was associated with a better outcome at discharge (mRS; *p* = .006), less need of mechanical ventilation (*p* = .028), shorter period of hospitalisation (*p* = .044), and lower rates of pneumonia (*p* = .007) and mortality (*p* = .011).

**Conclusion:**

Due to the inability of clinical assessments to detect silent aspiration, FEES might be better suited to identify stroke patients at risk and may contribute to a better functional outcome and lower rates of pneumonia and mortality. Our findings also point to a low awareness of dysphagia, even in a specialised stroke centre.

FEES in acute stroke patients helps to adjust the oral diet for the vast majority of stroke patients (69.1%) based on their swallowing abilities, potentially avoiding severe complications.

## Background

Dysphagia occurs in the course of many neurological diseases and frequently determines the outcome [1] with stroke being the most common cause. Up to 80% of stroke patients suffer from dysphagia, depending on the choice of diagnostics tests used (screening tests, comprehensive swallowing assessment by SLT and instrumented methods, such as VFS or FEES) [2]. Pneumonia due to dysphagia is the leading cause of death in stroke patients [3]. The risk for pneumonia increases up to 11.5-fold in stroke patients, if penetration or aspiration of secretions, food or fluids is present [2]. Hyperthermia, that can be caused by the pneumonia-associated fever, is known to be associated with a worse functional outcome in stroke [4]. Another known factor associated with a worse outcome in stroke patients is new or pre-existing malnutrition, which can also be caused by dysphagia [5]. Moreover, dysphagia is an independent predictor of disability and poor outcome, increased mortality, morbidity and markedly reduced quality of life in stroke [8–11]. Dysphagia not only leads to further complications in stroke patients, but its resultant long-term healthcare costs underline its socioeconomic relevance [6, 7].

Thus, one of the positive effects of stroke unit therapy seems to be an early diagnosis of dysphagia and the improvement of the swallowing function, thereby preventing pneumonia, malnutrition and dehydration.

Diagnostic tools for dysphagia include a screening examination, comprehensive swallowing examination (CSE) performed by physicians or speech and language therapists (SLT) as well as instrumented methods, such as videofluoroscopy of swallowing (VFS) or flexible endoscopic evaluation of swallowing (FEES). FEES combines many advantages in the clinical routine, as it is a bedside procedure without radiation exposure; enables the evaluation of saliva handling; it may be performed in uncooperative or unconscious patients; and it can easily be repeated. Moreover, swallowing is assessed by FEES in a more “natural” way than VFS, as the latter requires a contrast media and cued swallowing (to reduce radiation dosage). In this context, FEES might be an important tool for identifying patients at risk and may ultimately help improve functional outcome by adjusting patients’ oral diet.

We recently published a paper on the use of FEES and adjusting the oral diet in neurological patients. In this analysis, we were able to demonstrate a lower rate of pneumonia an a lower mortality, when adjusting the oral diet [12]. This is a subgroup analysis for acute stroke patients. As mentioned above, dysphagia is very common in stroke patients, puts those patients at a high risk of complications, increases mortality and leads to a longer length of hospitalisation. Furthermore, outcome parameters, such as the National Institute of Health Stroke Scale (NIHSS) and the modified Rankin-scale (mRS) are routinely assessed in all stroke patients. Therefore, the aim of the current study was to analyse the impact of FEES and adjustment of the oral diet based on those findings in the management of acute stroke patients. The study was designed as a cross-sectional hospital-based registry.

## Methods

**Fig. 1:**
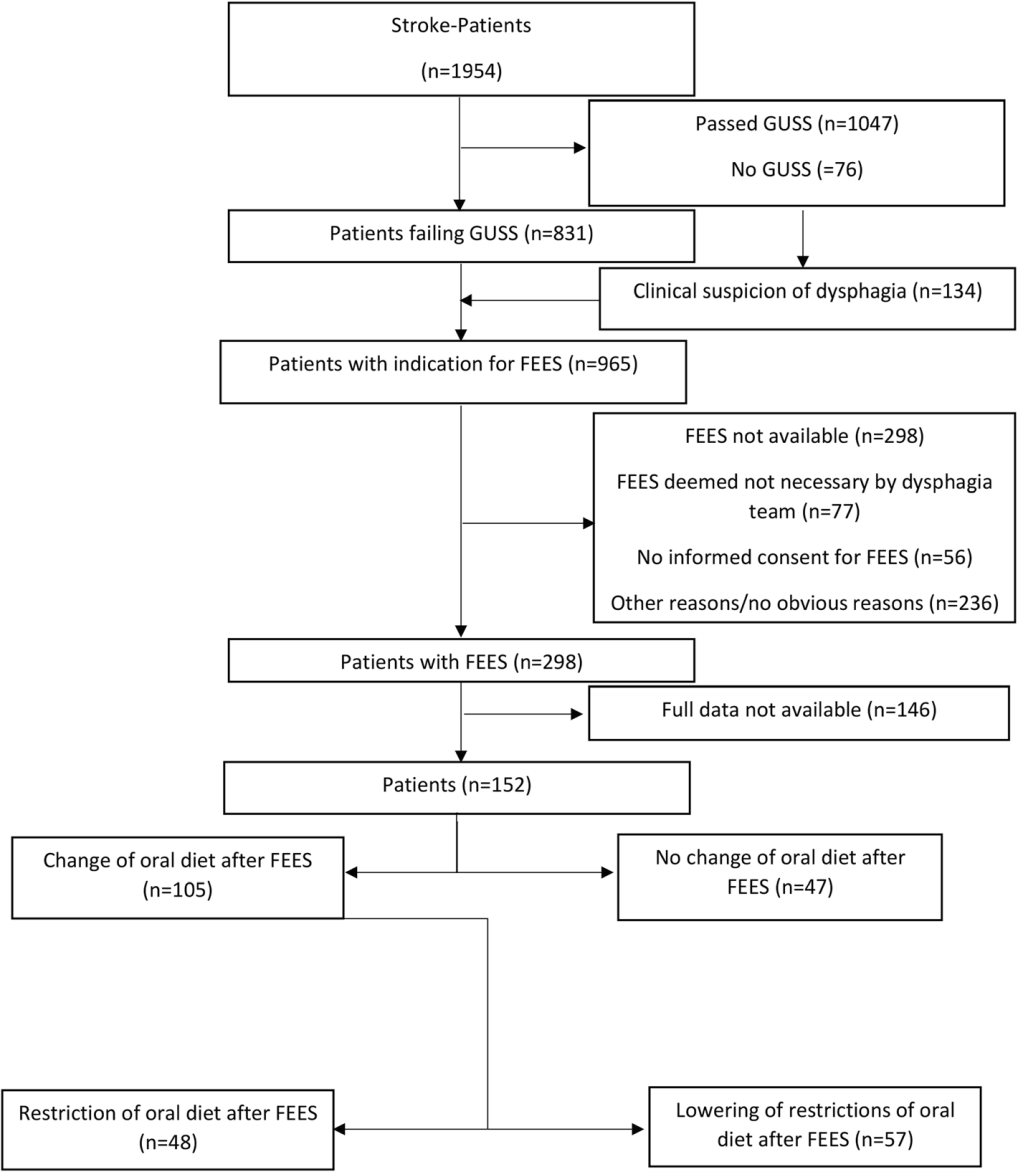
Screening process and decisions after FEES in patients

The study was done in a large German university hospital. As a part of routine care delivery for patients hospitalised for acute stroke, FEES was performed in case of a pathologic bedside screening procedure, performed by nurses or SLTs. In our department, we use the Gugging Swallowing Screen (GUSS) [13]. If the patient passed the GUSS, no FEES was performed and full oral diet was chosen. If the GUSS indicated possible dysphagia, the patient underwent a CSE by an SLT and FEES by a team consisting of a SLT and a neurologist. FEES was also performed if a patient showed signs of pharyngeal dysphagia during hospitalisation (e.g. wet voice, coughing when drinking, etc.) and if a patient developed signs of infection (productive cough, elevated inflammatory markers). The signs of dysphagia were reported by nurses, SLTs or the treating physicians. The patients with signs of dysphagia were discussed among the “dysphagia experts” of our department and indication for FEES was confirmed; oral diet prior to FEES was chosen as instructed by the GUSS or by clinical judgement of the treating physician. For quality control reasons, our findings gathered from examinations were documented systematically. All FEES were performed in a standardised manner by experienced physicians. The screening process is depicted in Fig. 1.

### Patients

All stroke patients treated in our department from January 2014 to September 2016 in whom FEES was performed were documented in a standardised manner. Approximately 800 patients per year are discharged from our hospital with the diagnosis of a stroke. Data documented in the database included age, sex, length of stay in hospital, stroke entity (ischemic stroke vs. primary haemorrhage), stroke aetiology (TOAST-criteria in ischemic stroke), NIHSS and mRS on admission and at discharge, localisation of ischemic lesion (left/right hemisphere, bilateral infarctions, brain stem), ischemic vascular territory, presence of risk factors in ischemic stroke (hypertension, atrial fibrillation, diabetes mellitus, hyperlipidaemia, tobacco smoking, cardiovascular disease, previous stroke), occurrence of pneumonia at any given point during hospitalisation (determined by the treating physician due to clinical signs of pneumonia, elevated inflammatory markers in the blood and chest X-ray), treatment on intensive care unit, necessity of intubation and mechanical ventilation lasting longer than 24 h (excluded from this item were patients intubated for surgery, such as decompressive craniotomy in cerebellar infarction or those who were preclinically intubated and promptly extubated), mortality, presence of dysphagia (as determined by the FEDSS, see below) and type of oral intake (before and after FEES; as determined by the FOIS, see below). For acquisition and use of data for scientific analyses, ethical approval was obtained from the local ethical committee (protocol number 208/16). All patients documented in the database were selected for the current analysis.

### Fees

FEES is a videoendoscopic nasolaryngeal swallowing study. We performed FEES following a standardised FEES® protocol, according to Langmore [14]: after applying decongestants (Xylometazolin) and local anaesthesia of the nasal duct using 2% Lidocaine-gel, a small endoscope (about 4 mm in diameter) was introduced through the inferior nasal meatus and the nasopharynx in the oropharynx. The swallowing of saliva and different consistencies of food and liquids and penetration, aspiration, localisation and extent of residues, as well as patients’ reactions (such as coughing), were visualised and documented. By definition, penetration is entering of any material into the airway above the level of the vocal folds, and aspiration is entering of any material below the level of the vocal folds [15]. In the first step of the procedure, anatomical changes, handling of saliva and the movement of swallowing-related structures were tested, then pudding-thick consistency (thickened water), normal water and solid food were introduced. For every consistency, we first used a teaspoon, then a table spoon and in case of water, the patient was asked to take a normal swallow from a cup. For better visualisation, the consistencies were dyed blue, using food colour. All consistencies were applied three times. If a consistence appeared unsafe to test, we skipped it; the consistence was rated as unsafe, if it entered the airway to the level of the vocal folds without ejection from the airway or any aspiration (score 5–8 on Rosenbek’s Penetration-Aspiration-Scale [16]). In the context of this research manuscript, we defined a “relevant dysphagia” as an oropharyngeal dysphagia with a score of 3–8 on Rosenbek’s Penetration-Aspiration-Scale, as this exposes the patient to the risk of pneumonia. Using the findings in FEES, the appropriate oral diet was chosen for the patient. Based on the pathophysiology found in FEES, compensatory and rehabilitative measures to treat dysphagia were carried out as described by Daniels and co-workers [17]. All FEES procedures were performed or supervised by an experienced investigator and lasted about 10 min each.

### Outcome measurements

Oral intake and dysphagia severity were measured by use of the functional oral intake scale (FOIS) and the Fiberoptic Endoscopic Dysphagia Severity Score (FEDSS), respectively:

FOIS is a seven-tiered scale ranging from 1 = no oral intake at all (NPO = nil per os) to 7 = full oral intake without restrictions (Table 5) [18]. For easier readability, the data of the functional oral intake scale were categorised in either NPO (FOIS = 1), tube dependency with at least some oral intake (FOIS 2–3), patients without tube dependency with dietary restrictions (single consistency, special preparations or limited specific food) (FOIS =4–6) and oral diet without restriction (FOIS = 7). Restriction of the oral diet was defined as a negative change on the FOIS, whereas lowering of restrictions of the oral diet was defined as a positive change. FOIS was documented prior to and after FEES.

There is no standardised way of defining the overall severity of dysphagia. In our department, we use the FEDSS-scale developed by Dziewas and co-workers [19]. The FEDSS is a six-tiered scale originally designed for use in stroke patients (Table 6). All parameters were recorded in a standardised way.

For evaluating the value of performing FEES in neurological patients, the following parameters were correlated with baseline data and dependent factors:

- Dysphagia as defined by a FEDSS score of ≥2
- The oral intake status as calculated by the FOIS and its overall change and type of change after FEES

### Statistical analyses

Absolute and relative frequencies were calculated based on cross-tables. For comparing relative frequencies, we used a two-tailed Fisher’s exact test. Continuous variables were analysed by calculating the median value and the interquartile range (IQR; 25% percentile and 75% percentile). Nonparametric non-paired data were analysed using the Mann–Whitney U-test and paired data using the Wilcoxon-test. Binary logistic regression analysis was used to identify factors associated with the item “change in oral diet”. All statistical analyses were performed using SPSS, release version 22.0 (SPSS©, Inc., IBM Company, 2015, Chicago-IL).

## Results

### Patients’ characteristics

173 FEES were performed in 152 stroke patients. In 19 (12.5%) patients, the procedure was repeated at least once and their data were only analysed once. In order to prevent data distortion, only the results of the first examination were included in the analysis of patients who received more than one FEES. 94 patients (61.8%) were male and the overall median age was 73 years (IQR 61.25–81 years). 119 patients were older than 60 years (78.3%). 125 patients (82.2%) were diagnosed with ischemic stroke and 27 (17.8%) with primary haemorrhage. 61 patients (48.8%) were treated on the intensive care unit. 62 patients (40.8%, or 26.8% when excluding intensive care patients) were diagnosed with pneumonia and 8 patients (5.3%) died during hospitalisation. Initially, 76 patients (49%) had no oral intake (NPO), 12 patients (7.8%) were tube dependent with at least some oral intake (FOIS 2–3), 45 patients (29.6%) needed no feeding tube but had dietary restrictions intake and 19 patients (12.3%) oral intake without restrictions. Among the patients with NPO or that were tube dependent with some oral intake, 65 patients (42.8%) had a nasogastric feeding tube prior to FEES and 2 patients (1.3%) had a PEG-tube. 31 patients (20.3%) needed intubation with a length of mechanical ventilation of 88 h (IQR 23–479; median 193 h [IQR 67.5–496.5], when excluding ventilation lasting less than 24 h). Of these patients, 11 were intubated preclinically and 13 during the first 6 h in our hospital. We were unable to reconstruct the reason for intubation in a sufficient number of patients from our data.

Patients’ characteristics are presented in Table 1. Patients’ characteristics for the subgroup of ischemic stroke patients can be found in the Additional file 1: Table S1.

**Table 1:** Baseline characteristics in stroke patients with normal swallowing function vs. patients with relevant dysphagia. Statistically significant p-levels are printed in bold

	Total cohort (*n* = 152)	Normal swallowing function (*n* = 42)	Relevant dysphagia (*n* = 110)	*P*
Sex				
Male	94 (61.8%)	25 (59.5%)	69 (62.7%)	0.427
Age median (IQR)	73 (61.25–81)	71 (58.5–80)	74 (63–81)	0.198
Stroke entity				
ischemic stroke	125 (82.2%)	34 (81%)	91 (82.7%)	
primary haemorrhage	27 (17.8%)	8 (19%)	19 (17.3%)	
Stroke severity on admission				
NIHSS on admission; median (IQR)	10 (5–15.5)	7 (4–12)	11 (6–17)	**0.013**
mRS on admission; median (IQR)	4 (3–5)	4 (3–5)	5 (4–5)	**0.012**
Stroke severity at discharge				
NIHSS at discharge; median (IQR)	6 (3–11)	4 (1–9.5)	7 (4–12)	**0.05**
mRS at discharge; median (IQR)	4 (3–5)	4 (2–4)	4 (3–5)	**0.002**
Time from admission to first FEES in days (median, IQR)	6 (3–11)	6 (2–10.25)	6 (3–11)	0.497
Length of stay in hospital in days (median, IQR)	17 (12–27.75)	15.5 (11.75–25.25)	18 (12–29)	0.225
Intensive care unit	61 (48.8%)	14 (33.3%)	47 (42.7%)	0.378
Necessity for intubation & mechanical ventilation lasting longer than 24 h	29 (19.1%)	4 (9.5%)	25 (22.7%)	**0.023**
Pneumonia	62 (40.8%)	16 (38.1%)	46 (41.8%)	0.715
Death	8 (5.3%)	0	8 (7.2%)	0.107
PEG procedure	34 (22.4%)	8 (19%)	26 (23.6%)	0.665
Diet after FEES				
No change in oral diet	47 (30.9%)	7 (16.7%)	40 (36.4%)	**0.019**
Change in oral diet	105 (69.1%)	35 (83.3%)	70 (63.6%)	**0.019**
Restriction	48 (31,6%)	1 (2.4%)	47 (42.7%)	**< 0.001**
Lowering of restrictions	57 (37,5%)	34 (81%)	23 (20.9%)	**< 0.001**

IQR: interquartile range

NIHSS: National institute of Health Stroke Scale

mRS: Modified Rankin-Scale

PEG: percutaneous endoscopic gastrotomy tube

### FEES examination

No side effects occurred, such as laryngospasm, syncope or epistaxis.

**Fig. 2:**
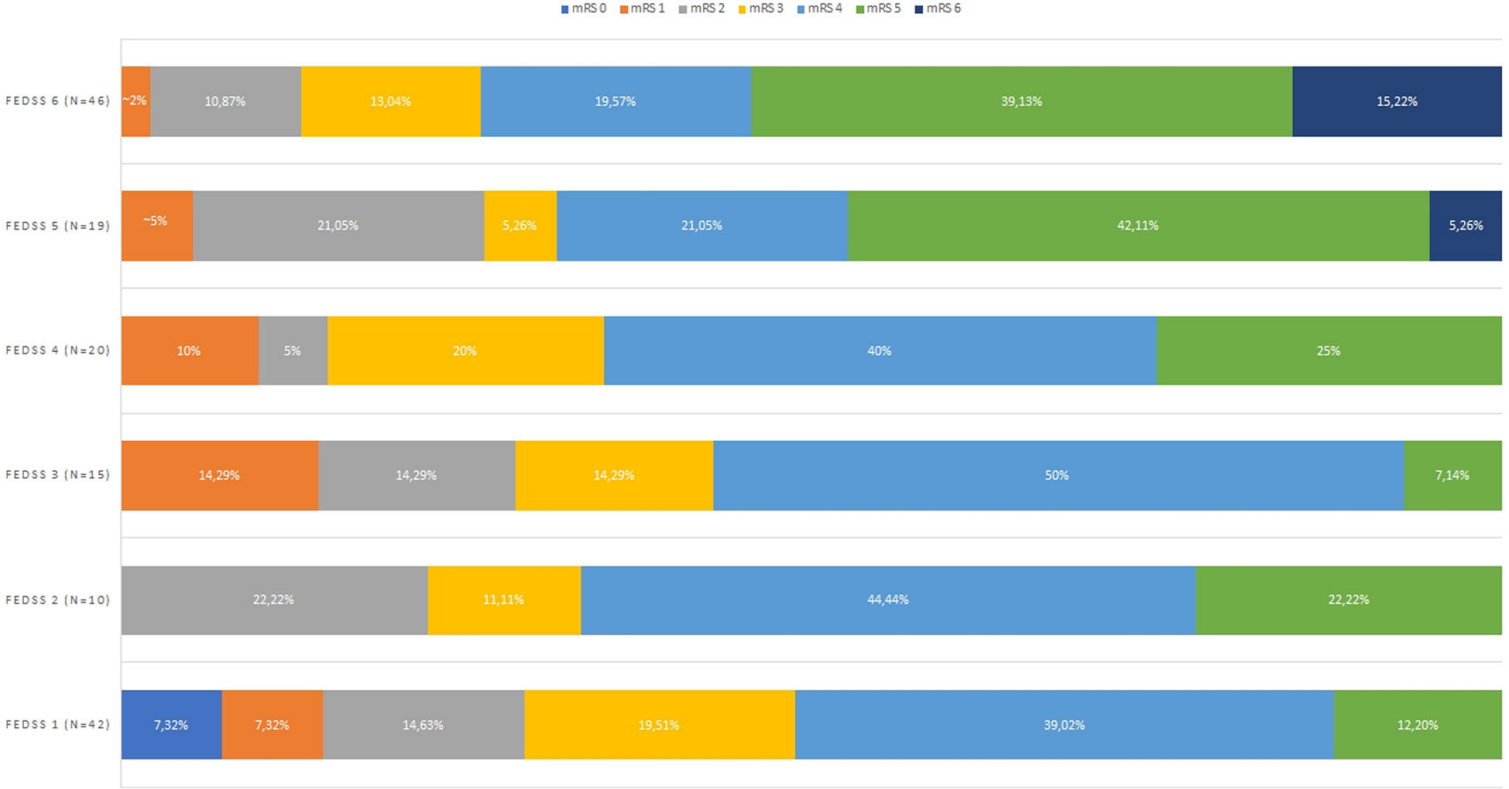
Outcome at discharge in relation to severity of dysphagia (FEDSS-Score)

The median FEDSS in the entire study population was 4 (IQR 1–6) and the median time from admission to first FEES was 6 days (IQR 3-11 days). FEES identified 110 (72.4%) patients with dysphagia (FEDSS 2–6). A diet modification was indicated in 105 patients (69.1%) with restriction of oral diet in 48 patients (31.6%) and lowering of restrictions in 57 (37.5%). NPO was indicated for the majority of patients (76.6% in this subgroup) without change in oral diet. 8 patients (5.3%) died during hospitalisation; all of them suffered from dysphagia. NIHSS and mRS on admission and at discharge were higher in dysphagic patients than non-dysphagic patients (admission: median NIHSS 11 [IQR 6–17] vs 7 [4–12], *p =* .013; median mRS 5 [IQR 4–5] vs. 4 [IQR 3–5], *p =* .012; discharge: median NIHSS 7 [IQR 4–12] vs 6 [3–11], *p =* .05; median mRS 4 [IQR 3–5] vs. 4 [IQR 2–4], *p =* .002). The outcome at discharge (mRS) in relation to the FEDSS is summarized in Fig. 2. Results are summarized in Table 1. The results for the subgroup of ischemic stroke patients can be found in the Additional file 1: Table S1.

### Differences in patients with and without change in oral diet

For patients that needed diet changes, the length of stay was shorter (median 16 days [IQR 11–25] vs. 22 days [IQR 13–30], *p =* .027), intubation and mechanical ventilation were less frequently indicated (15.2 vs. 31.9%, *p =* .028) and pneumonia as well as mortality rates were lower (pneumonia; 33.3% vs. 57.4%, *p =* .007; mortality: 1.9% vs 12.8%, *p =* .011). At discharge, mRS was lower in patients with diet changes (median 4 [IQR 3–4] vs. 4.5 [3–5], *p =* .006). A comparison of the intraindividual difference of mRS on admission and at discharge revealed a better functional outcome in patients with a change in oral diet (*p =* .001); we observed no better outcome in patients without a change in oral diet (*p =* .583). Results are summarised in Table 2 and Additional file 1: Table S2 (ischemic stroke patients only).

**Table 2:** Differences in baseline characteristics between stroke patients with and without change in the oral diet. Statistically significant p-levels are printed in bold

	Total cohort (n = 152)	No change in oral diet (*n* = 47)	Change in oral diet (*n* = 105)	*P*
Sex				
Male	94 (61.8%)	30 (63.8%)	64 (61%)	0.857
Age median (IQR)	73 (61.25–81)	75 (65–79)	72 (61–81.5)	0.657
Stroke entity				
ischemic stroke	125 (82.2%)	38 (80.9%)	87 (82.9%)	
primary haemorrhage	27 (17.8%)	9 (19.1%)	18 (17.1%)	
Stroke severity on admission				
NIHSS on admission; median (IQR)	10 (5–15.5)	11 (5.5–17.5)	9 (5–14)	0.237
mRS on admission; median (IQR)	4 (3–5)	5 (4–5)	4 (3–5)	0.087
Stroke severity at discharge				
NIHSS at discharge; median (IQR)	6 (3–11)	8 (3–13.5)	6 (3–10)	0.172
mRS at discharge; median (IQR)	4 (3–5)	4.5 (3–5)	4 (3–4)	**0.006**
Time from admission to first FEES	6 (3–11)	6 (3–13)	6 (2–10)	0.297
Length of stay in hospital in days (median, IQR)	17 (12–27.75)	22 (13–30)	16 (11–25)	**0.027**
Intensive care unit	61	22 (46.8%)	31 (29.5%)	**0.044**
Necessity for intubation & mechanical ventilation lasting longer than 24 h	31 (20.4%)	15 (31.9%)	16 (15.2%)	**0.028**
Pneumonia	62 (40.8%)	27 (57.4%)	35 (33.3%)	**0.007**
Death	8 (5.3%)	6 (12.8%)	2 (1.9%)	**0.011**
PEG procedure	34 (22.4%)	13 (27.7%)	21 (20%)	0.3

IQR: Interquartile range

NIHSS: National Institute of Health Stroke Scale

mRS: Modified Rankin-Scale

PEG: Percutaneous endoscopic gastrotomy tube

Binary logistic regression analysis revealed a lower odds-ratio associated with a change of oral diet for pneumonia (Table 3) and intubation (Table 4). The results for the subgroup of ischemic stroke patients can be found in Additional file 1: Table S3 and Table S4.

**Table 3:** Binaryl logistic regression analysis for pneumonia. Statistically significant p-levels are printed in bold

	*P*	Odds-Ratio	95%- Confidence interval
Age above 60	0.367	0.683	0.299–1.563
mRS on admission ≥3	0.897	0.936	0.342–0,256
Change of oral diet	**0.007**	0.362	0.173–0.754
Intubation	0.789	1.125	0.476–2.658
Constant	0.042

**Table 4:** Binary logistic regression analysis for intubation

	*P*	Odds-Ratio	95%- Confidence interval
Age above 60	0.511	0.716	0.264–1.940
mRS on admission ≥3	0.340	2.132	0.450–10.094
Change of oral diet	0.051	0.421	0.176–1.005
Pneumonia	0.808	1.113	0.470–2.633
Constant	< 0.001		

## Discussion

In 72% of our stroke patients, FEES unveiled a relevant dysphagia, leading to an adjustment of oral diet. In those patients, we observed a better functional outcome at discharge and fewer complications, such as the need for mechanical ventilation, a lower mortality rate and a lower rate of pneumonia.

The most alarming result is that only 30.9% of our patients had an appropriate oral diet for their swallowing abilities prior to FEES, meaning more than two thirds of our patients needed adjustment of their oral diet. This demonstrates low awareness of dysphagia and emphasises the need for instrumental diagnostics with a low threshold, proving that extensive clinical expertise avoids significant complications. Screening and CSE are necessary, but unable to detect all kinds of relevant swallowing disturbances, especially silent aspiration. FEES seems to be a more reliable tool than screening and CSE, as it revised the diet strategy suggested by screening and CSE in the majority of patients. Our results underline the necessity of performing FEES at a low threshold in the majority of stroke patients, irrespective of clinical examination and screening tests. This would be in accordance with intentions to change national guidelines as suggested by Lindner-Pfleghar and co-workers [20]. FEES is a safe, fast and reliable tool, as we observed no side effects in about 1730 min of examination. In our cohort, the median time from stroke to FEES was 6 days. As we found no side effects from FEES-examination and the patients’ diet was changed in the majority of patients, we recommend using FEES early in the acute phase of stroke unit treatment. Recently, a large FEES-registry study with various neurological diseases was published. The results of this study also confirm the safety of FEES, even when performed by an inexperienced investigator. In this study, the diet was adjusted in approximately about 50% of the patients after the FEES-examination [21].

When adjusting oral diet based on our findings in FEES, we observed in our patients a better outcome, a reduced need of intubation and mechanical ventilation, a lower pneumonia rate, lower mortality and a shorter period of hospitalisation. In a meta-analysis, Steele and co-workers reported a significant reduction in penetration and aspiration when thickening fluids [22]; this might be one factor explaining our results. One other factor contributing to a better outcome might be the increased mobility of patients after removal of a nasogastric feeding tube or an intravenous canulae for parenteral feeding, heightening the effects of physiotherapy. In case of a change in oral diet, the risk of pneumonia or intubation was reduced. Our findings underline the value of FEES in finding a safe oral diet for stroke patients.

Dysphagic patients had a higher NIHSS and mRS on admission and at discharge. This is in agreement with results by Dziewas and co-workers, who showed that patients with a NIHSS > 3 had signs of penetration and aspiration [19]. Warnecke and co-workers showed that the degree of dysphagia is predictive of functional outcome three months after the initial stroke [9]. Hence, the functional deficit seems to be predictive of dysphagia and vice versa.

Dysphagia was more often diagnosed in right hemispheric ischemia. Teismann and co-workers could visualize a time-dependent cortical activation of the hemispheres during swallowing using magnetencephalography; during the oral phase of swallowing there was a predominantly left-hemispheric activation, whereas right-sided activation was apparent during the pharyngeal phase of swallowing [23]. As we detected penetration and aspiration (occurring during the pharyngeal phase of swallowing) more often in our patients in right-hemispheric ischemia, our data are in accordance with these findings. Right-sided brain lesions are also associated with neglect and lack of awareness, disposing patients to aspiration [24, 25]. This might be an additional explanation for our findings.

As far as we know, only one study has been published on the effect of FEES about the functional outcome in stroke patients [26]. Bax and co-workers found a reduction in the pneumonia rate and a higher rate of a normal diet at discharge after FEES implementation than before the procedure. The length of stay in their study was longer than in ours, and there were no differences in mortality when FEES was performed routinely. However, this study had potential flaws: (i) the authors compared their patients with a historical control group; (ii) in both groups, the majority of patients were not examined via FEES; and (iii) in terms of functional outcome, the scores of the commonly used NIHSS and mRS at discharge were not reported. Thus, there is no evidence on the impact of FEES on the neurological functional outcome based on these study results.

Our study shows associations between adjusting the diet based on FEES findings and the functional neurological outcome, necessity for intubation and the rate of pneumonia and mortality. Our study design does not allow us to differentiate whether our results regarding better outcomes and fewer complications are based on our intervention (adjustment of oral diet based on FEES-findings) or fewer deficits of the patients. In our opinion, this effect could only be demonstrated by a randomised-controlled trial with patients receiving FEES or no FEES. As we have demonstrated in our cohort that more than two thirds of patients lacked an oral diet, that suited their swallowing abilities. It seems questionable to design a trial that withholds a potentially beneficial diagnostic test to one half of the study population. Potential selection bias of a large number of intensive care patients needs consideration when interpreting our results; as these patients are more severely affected by stroke, this explains the median mRS of 4. Our findings would therefore overestimate the number of neurological patients affected by dysphagia in this context, which might explain the high frequency of pneumonia compared to other studies [27]. Another circumstance to bear in mind when interpreting our results are the effects of rehabilitatory and compensatory strategies that were chosen based on FEES findings. Those strategies might also contribute to fewer complications and better patient outcomes, meaning the change in oral diet might not be the single factor for our results. However, all patients were treated by the same SLTs and when the patient was able to use those techniques, he or she was trained accordingly and instructed to use them. Therefore, the effects of rehabilitatory and compensatory techniques might impact our results. However, these effects should also be present in the group without change in oral diet, as they were also instructed to use these techniques. The long period of 6 days from admission to FEES can be mainly attributed to the large number of intensive care patients, as those patients could only undergo FEES after end of sedation, mechanical ventilation and extubation. These are the study’s main limitations; however, the study design represents the clinical routine with a pre-selection of patients by using a screening followed by an instrumented diagnostic. The study’s biggest limitation is the lack of a control group, reducing the validity of our results. Because of ethical reasons, we used no control group (without FEES), as - in our opinion - the risk of pneumonia and pneumonia-related death would have been too high. As discussed above, a randomised-controlled trial would be necessary, to clearly demonstrate the effects of FEES.

## Conclusions

FEES can better identify acute stroke patients at risk than screening for dysphagia or CSE due to its ability to detect silent aspiration. It is a safe and fast procedure that led to an adjustment of oral diet in roughly two out of three patients, with potential positive consequences for the overall clinical outcome by avoiding pneumonias or mechanical ventilation. Based on our data, and despite the need for large-scaled and randomised-controlled studies, we recommend the use of FEES in stroke patients at a low threshold.

### Supplementary information

**Additional file 1: Table S1.** Differences in baseline characteristics between patients with normal swallowing function versus those with clinically relevant dysphagia in the subgroup of patients with ischemic stroke. **Table S2.** Differences in baseline characteristics between stroke patients with and without change in oral diet in the subgroup of patients with ischemic stroke. **Table S3**. Binaryl logistic regression analysis for pneumonia in ischemic stroke patients. **Table S4**. Binary logistic regression analysis for intubation in ischemic stroke patients.

## Data Availability

The authors declare that the data supporting the findings of this study are available within the article. The data that support the findings of this study are not publically available due to local medical data protection policies.

## Appendix 1

**Table 5:** Functional oral intake scale [18]

1	Nothing by mouth (NPO)
2	Tube dependent with minimal attempts of food or liquid
3	Tube dependent with consistent oral intake of food or liquid
4	Total oral diet of a single consistency
5	Total oral diet with multiple consistencies, but requiring special preparation or compensations
6	Total oral diet with multiple consistencies without special preparation, but with specific food limitations
7	Total oral diet with no restrictions

## Appendix 2

**Table 6:** FEDSS-Score [19]

Score		Main findings
6	Handling of secretions/Saliva	Penetration or Aspiration
5	Puree consistency	Penetration/aspiration without or insufficient protective reflex
4		Penetration/aspiration with sufficient protective reflex
4	Liquids	Penetration/aspiration without or insufficient protective reflex
3		Penetration/aspiration with sufficient protective reflex
2	Soft solid food	Penetration/aspiration or massive residues in valleculae or piriforms
1		No penetration/aspiration and no more than mild to moderate residues in valleculae or piriforms

## Abbreviations

BSE: Bedside screening examination
CSE: Comprehensive swallowing examination
CT: Computed tomography
FEDSS: Fiber endoscopic dysphagia severity scale
FEES: Flexible endoscopic evaluation of swallowing
FOIS: Functional oral intake scale
GUSS: Gugging Swallowing Screen
ICU: Intensive care unit
IQR: Interquartile range
MRI: Magnetic resonance imaging
mRS: Modified Rankin-scale
NIHSS: National Institute of Health stroke scale
NPO: Nil per os (no oral intake)
PEG: Percutaneous endoscopic gastrostomy tube
SLT: Speech and language therapist
VFS: Videofluoroscopy of swallowing
