# Supplementary material for: Adjustment of oral diet based on flexible endoscopic evaluation of swallowing (FEES) in acute stroke patients: a cross-sectional hospital-based registry study
Source: BMC Neurol. 2019 Nov 12;19:282. doi: 10.1186/s12883-019-1499-8 (PMC6852733; doi:10.1186/s12883-019-1499-8)
Supplement: Supplementary file 1 — Additional file 1: Table S1. Differences in baseline characteristics between patients with normal swallowing function versus those with clinically relevant dysphagia in the subgroup of patients with ischemic stroke. Table S2. Differences in baseline characteristics between stroke patients with and without change in oral diet in the subgroup of patients with ischemic stroke. Table S3. Binaryl logistic regression analysis for pneumonia in ischemic stroke patients. Table S4. Binary logistic regression analysis for intubation in ischemic stroke patients. [file 12883_2019_1499_MOESM1_ESM.docx]

**Additional file 1: Table S1.** Differences in baseline characteristics between patients with normal swallowing function versus those with clinically relevant dysphagia in the subgroup of patients with ischemic stroke.

|  | **Ischemic stroke patients**  (n=125) | **Normal swallowing function**  (n=34) | **Relevant Dysphagia**  (n=91) | *P* |
| --- | --- | --- | --- | --- |
| Sex |  |  |  |  |
| Male | 76 (60.8%) | 21 (61.8%) | 55 (60.4%) | >0.999 |
| Age (median, IQR) | 75 (62.5-81.5) | 71 (58.5-80) | 75 (65-82) | 0.135 |
| Stroke aetiology |  |  |  |  |
| Large artery atherosclerosis | 32 (25.6%) | 8 (23.5%) | 24 (26.4%) | 0.821 |
| Cardioembolism | 48 (38.4%) | 15 (67.6%) | 33 (36.3%) | 0.536 |
| Small vessel disease | 14 (11.2%) | 1 (2.9%) | 13 (14.3%) | 0.110 |
| Other determined aetiology | 6 (4.8%) | 2 (5.9%) | 4 (4.4%) | 0.663 |
| Undetermined aetiology | 25 (20%) | 8 (23.5%) | 17 (18.7%) | 0.617 |
| Localisation of ischemic lesion |  |  |  |  |
| Left hemispheric (includes bilateral lesions) | 52 (41.6%) | 16 (47.1%) | 36 (39.6%) | 0.543 |
| Right hemispheric (includes bilateral lesions) | 50 (40%) | 9 (26.5%) | 41 (45.1%) | 0.066 |
| Bilateral | 10 (8%) | 3 (8.8%) | 7 (7.7%) | 0.705 |
| Brain Stem | 13 (10.4%) | 4 (11.8%) | 9 (9.9%) | 0.184 |
| Vascular territory |  |  |  |  |
| Arteria cerebri anterior | 1 (0.8%) | 0 | 1 (1.1%) | >0.999 |
| Arteria cerebri media | 87 (71.3%) | 20 (58.8%) | 67 (73.6%) | 0.121 |
| Arteria cerebri posterior | 1 (0.8%) | 1 (2.9%) | 0 | 0.27 |
| Arteria cerebri media + anterior | 3 (2.5%) | 1 (2.9%) | 2 (2.2%) | >0.999 |
| Vertebrobasilar | 24 (19.7%) | 9 (26.5%) | 15 (16.5%) | 0.209 |
| Multiple vascular territories | 6 (4.9%) | 2 (5.9%) | 4 (4.4%) | 0.661 |
| Risk factors |  |  |  |  |
| Hypertension | 109 (87.2%) | 27 (79.4%) | 82 (90.1%) | 0.135 |
| Artrial fibrillation | 41 (32.8%) | 10 (29.4%) | 31 (34.1%) | 0.674 |
| Diabetes mellitus | 32 (25.6%) | 14 (41.2%) | 18 (19.8%) | **0.021** |
| Hyperlipidaemia | 49 (39.2%) | 11 (32.4%) | 38 (41.8%) | 0.412 |
| Tobacco smoking* | 21 (16.8%) | 6 (17.6%) | 15 (16.5%) | >0.999 |
| Cardiovascular disease† | 30 (24%) | 6 (17.6%) | 24 (26.4%) | 0.356 |
| Previous stroke | 26 (20.8%) | 7 (20.6%) | 19 (20.9%) | >0.999 |
| Stroke severity on admission |  |  |  |  |
| NIHSS on admission; median (IQR) | 10 (5-16) | 6.5 (4-12) | 11 (6-17) | **0.017** |
| mRS on admission; median (IQR) | 4 (3-5) | 4 (3-5) | 4 (4-5) | **0.017** |
| Stroke severity at discharge |  |  |  |  |
| NIHSS at discharge; median (IQR) | 6 (3-21) | 3 (1-7.5) | 7 (4-13) | **<0.001** |
| mRS at discharge; median (IQR) | 4 (3-5) | 3 (2-4) | 4 (3-5) | **0.001** |
| Time from admission to first FEES | 6 (2-9.5) | 5.5 (2-9) | 6 (3-10) | 0.201 |
| Length of stay in hospital in days (median, IQR) | 16 (11.5-26) | 14 (10.75-18.75) | 18 (12-29) | 0.062 |
| Intensive care unit | 34 (27.2%) | 8 (23.5%) | 26 (28.6%) | 0.656 |
| Necessity for intubation & mechanical ventilation lasting longer than 24h | 21 (16.8%) | 3 (8.8%) | 18 (19.8%) | 0.184 |
| Pneumonia | 49 (39.2%) | 12 (35.3%) | 37 (40.7%) | 0.682 |
| Death | 4 (3.2%) | 0 | 4 (4.4%) | 0.574 |
| PEG procedure | 26 (20.8%) | 5 (14.7%) | 21 (23.1%) | 0.458 |
| Diet after FEES |  |  |  |  |
| No change in oral diet | 38 (30.4%) | 7 (20.6%) | 31 (34.1%) | 0.191 |
| Change in oral diet | 87 (69.6%) | 27 (79.4%) | 60 (65.9%) | 0.191 |
| Restriction | 44 (35.2%) | 1 (2.9%) | 43 (47.3%) | **<0.001** |
| Lowering of restrictions | 43 (34.4%) | 26 (76.5%) | 17 (18.7%) | **<0.001** |

FEDSS: Fiberendoscopic Dysphagia Severity Scale

IQR: Interquartile range

NIHSS: National Institute of Health Stroke Scale

mRS: Modified Rankin-Scale

PEG: Percutaneous endoscopic gastrotomy tube

* Current smoker, or quit within last 5 years

† Cardiovascular disease includes one of the following conditions: ischemic coronary artery disease, myocardial infarctions, peripheral artery occlusive disease, congestive heart failure and valvular disease.**Additional file 1: Table S2.** Differences in baseline characteristics between stroke patients with and without change in oral diet in the subgroup of patients with ischemic stroke.

|  | **Ischemic stroke patients** (n=125) | **No change in oral diet**  (n=38) | **Change in oral diet**  (n=87) | *p* |
| --- | --- | --- | --- | --- |
| Sex |  |  |  |  |
| Male | 76 (60.8%) | 22 (57.9%) | 54 (62.1%) | 0.694 |
| Age (median, IQR) | 75 (62.5-81.5) | 75 (65.75-79.5) | 74 (62-82) | 0.776 |
| Stroke aetiology |  |  |  |  |
| Large artery atherosclerosis | 32 (25.6%) | 10 (26.3%) | 22 (25.3%) | >0.999 |
| Cardioembolism | 48 (38.4%) | 18 (47.4%) | 30 (34.5%) | 0.23 |
| Small vessel disease | 14 (11.2%) | 1 (2.6%) | 13 (14.9%) | 0.062 |
| Other determined aetiology | 6 (4.8%) | 2 (5.3%) | 4 (4.6%) | >0.999 |
| Undetermined aetiology | 25 (20%) | 7 (18.4%) | 18 (20.7%) | >0.999 |
| Localisation of ischemic lesion |  |  |  |  |
| Left hemispheric (includes bilateral lesions) | 52 (41.6%) | 18 (47.4%) | 34 (39.1) | 0.436 |
| Right hemispheric (includes bilateral lesions) | 50 (40%) | 15 (39.5%) | 35 (40.2%) | >0.999 |
| Bilateral | 10 (8%) | 2 (5.3%) | 7 (8%) | 0.72 |
| brain stem | 13 (10.4%) | 3 (7.9%) | 10 (11.5%) | 0.753 |
| Vascular territory |  |  |  |  |
| Arteria cerebri anterior | 1 (0.8%) | 0 | 1 (1.1%) | >0.999 |
| Arteria cerebri media | 87 (71.3%) | 27 (71.1%) | 60 (69%) | >0.999 |
| Arteria cerebri posterior | 1 (0.8%) | 0 | 1 (1.1%) | >0.999 |
| Arteria cerebri media + anterior | 3 (2.5%) | 0 | 3 (3.4%) | 0.551 |
| Vertebrobasilar | 24 (19.7%) | 8 (21.1%) | 16 (18.4%) | 0.809 |
| Multiple vascular territories | 6 (4.9%) | 3 (7.9%) | 3 (3.4%) | 0.374 |
| Risk factors |  |  |  |  |
| Hypertension | 109 (87.2%) | 35 (92.1%) | 74 (85.1%) | 0.387 |
| Artrial fibrillation | 41 (32.8%) | 16 (42.1%) | 25 (28.7%) | 0.153 |
| Diabetes mellitus | 32 (25.6%) | 10 (26.3%) | 22 (25.3%) | >0.999 |
| Hyperlipidaemia | 49 (39.2%) | 17 (44.7%) | 32 (36.8%) | 0.431 |
| Tobacco smoking* | 21 (16.8%) | 5 (13.2%) | 16 (18.4%) | 0.606 |
| Cardiovascular disease† | 30 (24%) | 14 (36.8%) | 16 (18.4%) | **0.039** |
| Previous stroke | 26 (20.8%) | 7 (18.4%) | 19 (21.8%) | 0.812 |
| Stroke severity on admission |  |  |  |  |
| NIHSS on admission; median (IQR) | 10 (5-16) | 11 (6-17.75) | 9 (4-14.25) | 0.124 |
| mRS on admission; median (IQR) | 4 (3-5) | 5 (4-5) | 4 (3-5) | **0.048** |
| Stroke severity at discharge |  |  |  |  |
| NIHSS at discharge; median (IQR) | 6 (3-21) | 9 (3-14) | 5 (2-10) | 0.78 |
| mRS at discharge; median (IQR) | 4 (3-5) | 4 (3-5) | 4 (2-4) | **0.034** |
| Time from admission to first FEES | 6 (2-9.5) | 6 (2-9) | 6 (2-9) | 0.267 |
| Length of stay in hospital in days (median, IQR) | 16 (11.5-26) | 20 (12-30) | 15 (11-22) | **0.013** |
| Intensive care unit | 34 (27.2%) | 14 (36.8%) | 20 (23%) | 0.129 |
| Necessity for intubation & mechanical ventilation lasting longer than 24h | 21 (16.8%) | 9 (23.7%) | 12 (13.8%) | 0.198 |
| Pneumonia | 49 (39.2%) | 21 (55.3%) | 28 (32.2%) | **0.018** |
| Death | 4 (3.2%) | 2 (5.3%) | 2 (2.3%) | 0.584 |
| PEG procedure | 26 (20.8%) | 9 (23.7%) | 17 (19.5%) | 0.636 |

IQR: Interquartile range

NIHSS: National Institute of Health Stroke Scale

mRS: Modified Rankin-Scale

PEG: Percutaneous endoscopic gastrotomy tube

* Current smoker, or quit within last 5 years

†Cardiovascular disease includes one of the following conditions: ischemic coronary artery disease, myocardial infarctions, peripheral artery occlusive disease, congestive heart failure and valvular disease.

**Additional file 1: Table S3** – Binaryl logistic regression analysis for pneumonia in ischemic stroke patients

|  | ***P*** | **Odds-Ratio** | **95%- Confidence interval** |
| --- | --- | --- | --- |
| Age above 60 | 0.550 | 0.756 | 0.303 – 1.888 |
| mRS on admission ≥3 | 0.989 | 0.993 | 0.328 – 3.002 |
| Change of oral diet | **0.023** | 0.396 | 0.177 – 0.882 |
| Intubation | 0.32 | 1.327 | 0.498 – 3.537 |
| Constant | 0.02 |  |  |

**Additional file 1: Table S4** – Binary logistic regression analysis for intubation in ischemic stroke patients

|  | ***P*** | **Odds-Ratio** | **95%- Confidence interval** |
| --- | --- | --- | --- |
| Age above 60 | 0.956 | 1.035 | 0.307 – 3.484 |
| mRS on admission ≥3 | 0.273 | 3.236 | 0.397 – 26.371 |
| Change of oral diet | 0.34 | 0.614 | 0.226 – 1.671 |
| Pneumonia | 0.572 | 1.326 | 0.498 – 3.535 |
| Constant | <0.001 |  |  |
